# An Adaptive Spatio-Temporal Traffic Flow Prediction Using Self-Attention and Multi-Graph Networks

**DOI:** 10.3390/s25010282

**Published:** 2025-01-06

**Authors:** Basma Alsehaimi, Ohoud Alzamzami, Nahed Alowidi, Manar Ali

**Affiliations:** 1 Department of Computer Science, King AbdulAziz University, Jeddah 21589, Saudi Arabia; ualzamzami@kau.edu.sa (O.A.); nalowidi@kau.edu.sa (N.A.); mali@kau.edu.sa (M.A.); 2 Applied College, Taibah University, Madinah 41477, Saudi Arabia

**Keywords:** traffic flow prediction, temporal convolutional network, graph convolution network, graph attention networks, attention mechanism

## Abstract

Traffic flow prediction is a pivotal element in Intelligent Transportation Systems (ITSs) that provides significant opportunities for real-world applications. Capturing complex and dynamic spatio-temporal patterns within traffic data remains a significant challenge for traffic flow prediction. Different approaches to effectively modeling complex spatio-temporal correlations within traffic data have been proposed. These approaches often rely on a single model to capture temporal dependencies, which neglects the varying influences of different time periods on traffic flow. Additionally, these models frequently utilize either static or dynamic graphs to represent spatial dependencies, which limits their ability to address complex and overlapping spatial relationships. Moreover, some approaches struggle to fully capture spatio-temporal variations, leading to the exclusion of critical information and ultimately resulting in suboptimal prediction performance. Thus, this paper introduces the Adaptive Spatio-Temporal Attention-Based Multi-Model (ASTAM), an architecture designed to capture spatio-temporal dependencies within traffic data. The ASTAM employs multi-temporal gated convolution with multi-scale temporal input segments to model complex non-linear temporal correlations. It utilizes static and dynamic parallel multi-graphs to facilitate the modeling of complex spatial dependencies. Furthermore, this model incorporates a spatio-temporal self-attention mechanism to adaptively capture the dynamic and long-term spatio-temporal variations in traffic flow. Experiments conducted on four real-world datasets reveal that the proposed architecture outperformed 13 baseline approaches, achieving average reductions of 5.0% in MAE, 13.28% in RMSE, and 6.46% in MAPE across four datasets.

## 1. Introduction

Traffic prediction has evolved into a key aspect of advancing smart cities and is foundational to Intelligent Transportation Systems (ITSs) [1]. Different real-world applications have demonstrated the value and necessity of accurate traffic prediction, including urban infrastructure construction [2], traffic congestion [3], travel time estimation [4], taxi dispatching [5], pedestrian safety [6], planning and navigating routes [7], and urban transportation networks [8]. Therefore, improving the accuracy of traffic flow prediction has attracted significant attention from researchers.

The objective of traffic prediction is to predict future traffic conditions by analyzing historical traffic data and spatio-temporal data collected from sensors deployed across road networks. Unlike traditional time series analysis, traffic prediction is a challenging task that involves both temporal and spatial dependencies. Temporal dependencies are observed in the influence of traffic patterns across different times, while spatial dependencies are reflected in the interactions between traffic flows on different roads within the network. Furthermore, traffic prediction must consider the non-linear and dynamic nature of traffic conditions between sensors over time, including distinct recurring patterns such as peak and off-peak hour variations and weekday versus weekend differences. Accurately predicting traffic flow is therefore of great importance for mitigating traffic congestion, improving urban traffic conditions, and enhancing the quality of residents’ lives.

In recent years, researchers have increasingly incorporated Graph Neural Networks (GNNs) with Recurrent Neural Networks (RNNs) [9,10,11,12] for traffic prediction. GNNs [13] are employed to model the spatial dependencies within traffic road networks in non-Euclidean spaces. GNNs excel at handling the complexities of graph-structured data and effectively capturing the inherent spatial relationships within traffic data, making them ideal for traffic flow prediction tasks [14,15]. They directly input traffic signals into the model, utilizing an adjacency matrix to depict the connectivity between road segments [10,16,17,18]. RNNs [19] and their variations, such as Gated Recurrent Units (GRUs) [20] and Long Short-Term Memory (LSTM) [21], have been utilized to model the temporal correlations of traffic data due to their effectiveness in capturing short-term dependencies [22,23]. These GNN-RNN hybrid approaches offer a powerful method for traffic flow prediction that effectively captures spatio-temporal dependencies.

Despite the notable advancements and significant accomplishments achieved in the area of traffic prediction, existing studies still face some challenges that need to be tackled. Among these challenges is the inability to effectively capture the complex and dynamic spatial and temporal correlations inherent in traffic data.

For representing the temporal dependencies, many studies have relied solely on a single model, specifically RNNs, to capture temporal correlations in traffic prediction. However, traffic patterns are influenced not only by short-term intervals but also by consistent trends across different periods, such as hourly, daily, and weekly cycles. For instance, morning rush hour patterns on workdays differ significantly from those on weekends, and traffic levels at one time often correlate with recent times. While RNNs are effective for short-term dependencies, they struggle to fully capture the complex long-term patterns in traffic flow, which are crucial for accurate traffic prediction. Moreover, RNNs face challenges such as increased computational complexity, time-consuming iterations, and gradient issues when modeling these longer temporal dependencies. As a result, relying solely on single models is inadequate for fully addressing the temporal dependencies inherent in traffic data.

With regards to the spatial correlations, many existing studies depend on pre-defined static graphs that rely on distance to determine the relationships between road segments. However, in real-world situations, non-adjacent nodes can have strong relationships, and nodes with similar distances may not necessarily exhibit strong spatial dependencies due to hidden factors such as intersections, opposing lanes, or roadway closures. Moreover, spatial relationships are dynamic and subject to change over time due to non-linear correlations and unpredictable events like festivals, accidents, and road maintenance. These events lead to unexpected changes in traffic flow within adjacent areas. Therefore, models that rely on pre-defined graphs and ignore hidden and dynamic factors frequently struggle to capture the intricate spatial relationships within a road network, resulting in inaccurate traffic flow predictions.

Moreover, as the spatio-temporal data vary among both the temporal and spatial dimensions, traffic flow patterns differ by time and location. For instance, commercial areas typically experience higher traffic levels during the daytime compared to the evening time. Similarly, areas in commercial districts usually exhibit higher traffic overall, whereas residential areas tend to have lighter traffic. Therefore, it is crucial to consider the real-node characteristics in terms of both time and space scales to improve performance by employing a self-attention mechanism.

Motivated by the limitations mentioned above, this paper introduces an innovative architecture called the Adaptive Spatio-Temporal Attention-Based Multi-Model (ASTAM) for traffic flow prediction. This architecture employs multi-temporal gated convolution to capture temporal dependencies at various time periods. Additionally, a multi-graph approach is used to model complex spatial correlations, including an adaptive graph to capture hidden correlations and a dynamic graph for capturing dynamic spatial relationships between adjacent areas. Moreover, the architecture adaptively models the complex spatio-temporal variations by adopting a self-attention mechanism. This paper aims to address the following research question:

- ${RQ}_{1}$: To what extent does the proposed ASTAM architecture enhance the accuracy of traffic flow prediction?

To test this question, the following hypothesis is proposed:

- $H_{1}$: The proposed ASTAM architecture significantly enhances the accuracy of traffic flow prediction by effectively capturing complex spatio-temporal dependencies in traffic flow data.

To address the research question, the following key contributions are presented:

- A novel spatio-temporal multi-model architecture is proposed. This architecture integrates temporal gated convolution, an adaptive graph, a dynamic attention graph, and self-attention mechanisms. This unified approach leverages the strengths of each model to capture the complex spatio-temporal dynamics of traffic flow effectively.
- Multi-temporal gated convolutions are used to capture various time periods simultaneously and to represent temporal dependencies effectively, thereby enhancing prediction performance.
- A parallel multi-graph fusion is proposed that integrates an adaptive graph and a dynamic attention graph to accurately represent both hidden static and dynamic spatial dependencies among different road segments.
- A spatio-temporal self-attention mechanism is incorporated to adaptively capture spatio-temporal variation in the traffic flow.
- Extensive comparative experiments were conducted using 13 baselines across four publicly available datasets. The results demonstrate that the proposed architecture outperforms all baseline approaches. Additionally, ablation studies were performed to evaluate the distinct contribution of each component within the architecture.

The remaining sections of this paper are structured as follows: Section 2 reviews the relevant studies on traffic prediction. Section 3 presents the problem statement. Section 4 provides a detailed description of ASTAM. Section 5 presents a comprehensive comparison of ASTAM with 13 baseline methods across four real-world public datasets, as well as ablation studies and parameter sensitivity assessments. Section 6 discusses potential real-world applications of ASTAM. Section 7 summarizes and concludes this research.

## 2. Related Work

Traffic prediction is a major issue in intelligent urban development, yet it presents challenges due to the complex and dynamic spatio-temporal patterns within traffic data. The current approaches to traffic prediction are categorized into three main groups: statistical, traditional machine learning, and deep learning approaches.

Before the emergence of deep learning, early research employed statistical methods, including Vector Auto-Regression (VAR) [24] and an Autoregressive Integrated Moving Average Model (ARIMA) [25] for traffic prediction. Nevertheless, these statistical approaches are constrained by strict theoretical assumptions, reliance on previous knowledge, and fundamental statistical mathematics, which inadequately address the non-linear and complex nature of traffic patterns. Consequently, these approaches face difficulties in delivering precise predictions of traffic flow.

Traditional machine learning approaches, including Support Vector Regression (SVR) [26] and Support Vector Machines (SVMs) [27], have demonstrated substantially better accuracy than statistical techniques as they effectively capture non-linear correlations in traffic data. Despite their effectiveness, these approaches heavily rely on sophisticated mathematical techniques and manual feature engineering that limit their ability to effectively extract the intricate spatio-temporal relationships within the traffic data. Consequently, their performance may be constrained and challenging to improve further.

With data growing substantially in volume, deep neural networks have the capability to deliver superior performance in comparison to conventional time series analytical techniques when dealing with regression problems. Deep learning has demonstrated promising outcomes across various domains, including those that encompass traffic prediction, owing to their ability to model complex non-linear characteristics in spatio-temporal data [28]. RNNs and Convolutional Neural Networks (CNNs) are two methods that have been extensively employed in traffic prediction tasks.

Traffic flow prediction is considered a conventional time series prediction task; therefore, it is critical to capture the temporal dependencies. Various traffic prediction approaches use RNNs [19] and their variations, GRU [20] and LSTM [21], as temporal feature models to enhance the accuracy of traffic prediction [22,23]. However, when RNNs are the only models that are used to capture the temporal dependencies in the traffic data, the spatial dependencies between the road segments inherent in traffic patterns can be neglected.

To address the challenges faced by RNNs, spatio-temporal modeling approaches that rely on CNNs have been developed and extensively utilized in traffic prediction tasks for modeling the spatial relationships between different regions. In [29], the traffic data were represented as a sequence of images, and CNNs were applied to represent the spatial correlations. The authors in [30] introduced Deep Spatio-Temporal Residual Networks (ST-ResNets) which employ CNNs for spatial feature extraction in the traffic network. By stacking layers of RNNs and CNNs, the prediction accuracy was significantly enhanced. In [31,32], LSTM layers were employed alongside the CNN structure, which enables the seamless extraction of both spatial and temporal correlations. However, CNNs are typically limited to grid-like data, and modeling traffic data as a grid can result in the loss of valuable spatial information, leading to inaccurate predictions. The irregular placement of traffic sensors further demonstrated the inadequacy of CNNs for handling non-Euclidean data like traffic networks.

In recent years, researchers have investigated the use of GNNs to model traffic networks in non-Euclidean spaces. GNNs have proven their effectiveness in handling the complexities of graph-structured data and capturing the spatio-temporal relationships within those data [14,15]. Specifically, Graph Convolutional Networks (GCNs) [33] and Graph Attention Networks (GATs) [34] are utilized to extract spatial correlations in traffic data. Several studies have integrated GCNs and GATs with different RNN architectures to enhance traffic prediction. For example, a study [9] introduced a diffusion convolutional recurrent neural network (DCRNN) that employs bi-directional random walks in the graph for spatial dependency extraction, and GRUs are utilized for temporal correlation capturing. In [18], a novel Temporal Graph Convolutional Network (T-GCN), which integrates GCNs and GRUs to enhance traffic prediction performance, is introduced. Additionally, in [35], the GAT is employed to extract complex spatial dependencies, and a GRU model is utilized to extract temporal features. However, RNN architectures struggle to effectively capture long temporal patterns, leading to high computational complexity and extended processing times.

Rather than adopting RNN approaches for temporal correlation extraction, several studies have employed Temporal Convolutional Networks (TCNs) to capture the short-term and long-term temporal dependencies within traffic networks [36,37,38]. For instance, the authors in [10] proposed a Spatio-Temporal Graph Convolutional Network (STGCN) that uses the GCN to extract spatial correlations and employed convolutional structures to capture temporal dependencies. The study in [16] adopts a gated mechanism for TCNs to effectively extract complex temporal dependencies. This mechanism consists of a stack of spatio-temporal layers where an integration of GCNs and gated TCNs is used to capture spatio-temporal correlations of traffic data. However, the models in [10,16] excessively rely on handling pre-defined graphs that are based on Euclidean distances. Therefore, an Adaptive Graph Convolutional Recurrent Network (AGCRN) is proposed in [11]. The AGCRN avoids relying on pre-defined graphs by designing an embedding for each node to create an adaptive graph that can extract the hidden spatial relationships.

Additionally, attention mechanisms have been employed effectively to extract and emphasize crucial information, thereby improving the modeling of complex spatio-temporal relationships in traffic networks. An Attention-Based Spatio-Temporal Graph Convolutional Network (ASTGCN) model is introduced in [39]. This method integrates GCNs and CNNs to jointly extract dynamic spatio-temporal dependencies in traffic flow. The final prediction is obtained by weighting the outputs derived from three distinct temporal features, enhancing the capability of the model to represent sophisticated patterns within traffic data. Moreover, an advanced spatio-temporal attention mechanism was designed to effectively capture the dynamic dependencies among nodes, leading to the development of the Dynamic Spatial–Temporal-Aware Graph Neural Network (DSTAGNN) [40]. This model incorporates the GCN, with spatial weights, which are adaptively adjusted through an enhanced self-attention mechanism. Several studies, such as [41,42], have employed node-level attention mechanisms to adaptively model spatio-temporal variations by taking into account the real characteristics of nodes. These investigations have demonstrated that this technique is effective for enhancing traffic prediction tasks, primarily owing to its ability to extract the intricate correlations inherent in traffic data.

Beyond the previously discussed techniques, alternative methods including Dynamic Time Warping (DTW) [43], differential equations [44,45,46], and Gaussian function [47], have been employed to enhance traffic flow prediction and have led to significant outcomes. These approaches offer valuable perspectives on extracting the spatio-temporal relationships, thereby improving traffic prediction.

## 3. Preliminaries

**Definition 1.**  *(Road Traffic Network): This research represents the road traffic network as a graph G=(V,E,A), where*

- *$V=\{v_{1},v_{2},\ldots ,v_{N}\}$ is the set of traffic sensor nodes with |V|=N,N being the total sensor nodes in the graph G. They are installed at different intersections across the road traffic network to gather traffic data.*
- *E denotes set of edges connecting the node pairs in the graph G.*
- *$A\in R^{N\times N}$ is the weighted adjacency matrix of the traffic graph G, and is normalized using the following equation:*

(1) $$A_{i,j}=\left\{\begin{array}{cc} \exp\left(-{\left(\frac{dis(i,j)}{\sigma }\right)}^{2}\right) & \mathrm{if}\;\mathrm{dis}(i,j)\neq 0\\ 0 & otherwise \end{array}\right.$$

*where σ is the parameter for standard deviation that controls the rate of decay. dis(i,j) is the distance between nodes $v_{i},v_{j}\in V$. This exponential normalization assigns higher weights to closer nodes and lower weights to distant nodes. The normalization ensures that all values in the adjacency matrix are between 0 and 1.*

**Definition 2.**  *(Traffic Signal): $X_{t}=\left\{X_{t}^{1},X_{t}^{2},\ldots ,X_{t}^{N}\right\}$, where $X_{t}\in R^{N\times F},$ denotes the value collected from each sensor node in the graph G at given time t. F represents feature numbers for each sensor node in the graph G. These features could be volume, speed, and occupancy. N is the total sensor nodes in G.*

A traffic flow prediction task involves predicting future traffic patterns that have been inferred from historical observations over the past *T* time steps, denoted as $\left(X_{t-T+1},X_{t-T+2},\ldots ,X_{t-1},X_{t}\right)\in R^{N\times F\times T}$. The objective involves learning a non-linear function f(·) that precisely predicts future traffic flow over the following $T_{p}$ time steps. This could be formulated mathematically as follows:

(2) $$\left(X_{t-T+1},X_{t-T+2},\ldots ,X_{t-1},X_{t}\right)\overset{f(\cdot )}{⟶}\hat{X}=\left(X_{t+1},X_{t+2},\ldots ,X_{t+T_{p}-1},X_{t+T_{p}}\right)$$

## 4. Methodology

This paper introduces a novel architecture, the Adaptive Spatio-Temporal Attention-Based Multi-Model (ASTAM), which is designed to enhance ITS through precise traffic flow prediction by efficiently extracting spatio-temporal relationships in traffic data. This section outlines in detail the implementation of the proposed architecture.

### 4.1. The Architecture of the ASTAM

**Figure 1:**
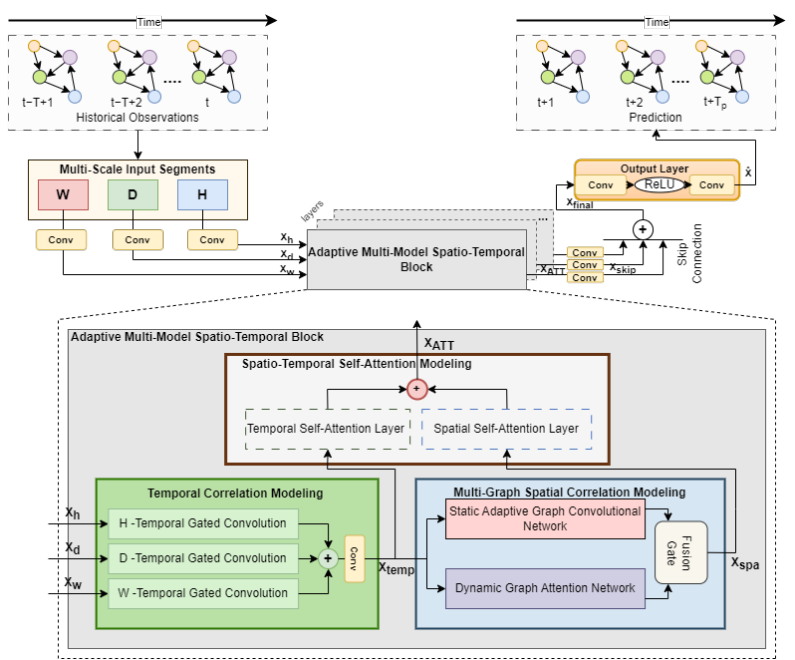
The comprehensive architecture of the ASTAM.

The ASTAM comprises multi-scale temporal input segments, a stacked Adaptive Multi-Model Spatio-Temporal block (AMST), and an output layer as depicted in Figure 1. The ASTAM processes historical time steps, represented as $(X_{h}$,$X_{d}$,$X_{w}$), and predicts future traffic flow for a specified duration, denoted by $T_{p}$.

The AMST includes three components: Temporal Correlation Modeling (TCM), Multi-Graph Spatial Correlation Modeling (MGSCM), and Spatio-Temporal Self-Attention Modeling (STSAM). The subsequent sections provide a detailed explanation for each of these components within the ASTAM.

### 4.2. Multi-Scale Temporal Input Segments

The sliding time window approach is employed to extract three different historical time segments: hourly, daily, and weekly, denoted by $X_{h}$, $X_{d}$, and $X_{w}$ with the lengths of the historical time steps as $T_{h}$, $T_{d}$, and $T_{w}$, respectively. Having three defined segments allows the model to integrate information from various time scales, which is why it can extract the complex temporal patterns inherent within traffic data effectively.

**Figure 2:**
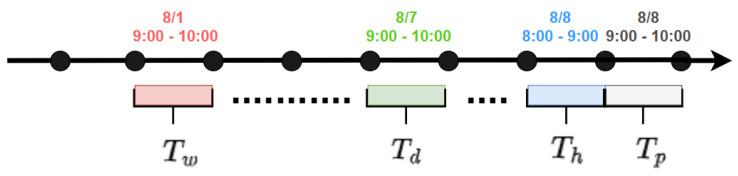
Creating multi-scale temporal input segments based on hourly, daily, and weekly cycles.

The hourly segment comprises the historical traffic data for the previous $T_{h}$ hours, representing the immediate past that likely influences the future traffic flow. As depicted in Figure 2, the hourly segment is indicated by the blue portion of the timeline and is defined as follows:

(3) $$X_{h}=\left(X_{t-T_{h}+1},X_{t-T_{h}+2},\ldots \ldots ,X_{t}\right)\in R^{N\times F\times T_{h}}$$

The daily-periodic segment comprises historical traffic data from the corresponding hours of previous $T_{d}$ days. These data are used to identify recurring daily patterns in traffic flow, where *s* is the sampling frequency per day. As illustrated in Figure 2, the daily segment is visually represented by the green portion of the timeline and is defined as follows:

(4) $$X_{d}=\left(X_{t-(T_{d}/T_{p})*s+1},\ldots ,X_{t-\left(T_{d}/T_{p}\right)*s+T_{p}},\ldots ,X_{t-s+1},\ldots ,X_{t-s+T_{p}}\right)\in R^{N\times F\times T_{d}}$$

The weekly-periodic segment comprises historical data from the same time of day and the same day of the past weeks to detect recurring weekly traffic patterns. As shown in Figure 2, the weekly segment is characterized by the red portion of the timeline and is defined as follows:

(5) $$X_{w}=\left(X_{t-7*(T_{w}/T_{p})*s+1},\ldots ,X_{t-7*\left(T_{w}/T_{p}\right)*s+T_{p}},\ldots ,X_{t-7*s+1},\ldots ,X_{t-7*s+T_{p}}\right)\in R^{N\times F\times T_{w}}$$

These input segments, consisting of historical time steps $(X_{h}$, $X_{d}$, $X_{w}$) ∈$R^{N\times F\times T}$, are initially projected into a high-dimensional space through linear transformations and then passed to the AMST. Specifically, input segments are passed to the TCM model.

### 4.3. Temporal Correlation Modeling (TCM)

The TCN is utilized with causal convolutions to model temporal correlations in traffic data. Dilated causal convolutions enable an exponentially expanding receptive field through increased layer depth, allowing the filter to skip input values at specific intervals. Unlike RNN-based methods, the TCN handles long-range sequences non-recursively, which enables parallel computation and prevents gradient vanishing. Zero-padding is applied to maintain the causal sequence, which guarantees that predictions made during each time step depend solely on past data. In each time step *t*, the temporal convolution mechanism *F* applied to input elements *s*, s∈X, filter **f**:{0,1,……,k} is calculated as follows:

(6) $$F\left(s\right)=X⋆f\left(t\right)\sum_{s=0}^{k-1}f\left(s\right)X\left(t-D\times s\right)$$

where *X* signifies input, f refers to the filter, ⋆ denotes the convolution operator, *k* represents convolution kernel size, and *D* is the dilation factor that determines the skipping distance.

**Figure 3:**
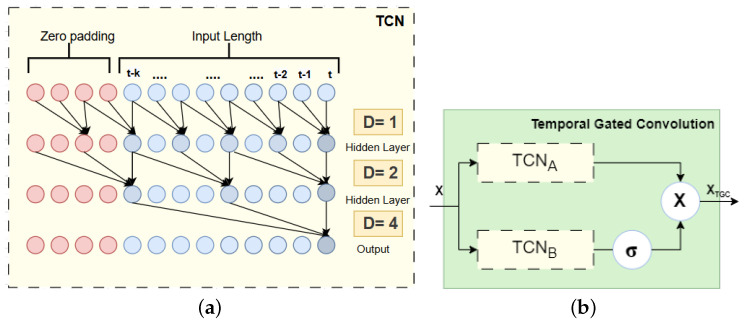
(**a**) TCN architecture illustration. (**b**) A structural layout of TGC.

As illustrated in Figure 3a, $D=2^{i-1}$ at the *i*th layer. The first layer has a dilation factor of D=1, performing a regular convolution without skipping any values. In the second layer, with D=2, the filter skips every second value during convolution. At the third layer, where D=4, the filter skips three values between convolutions. By increasing *D* in this manner, the model’s receptive field grows exponentially, enabling it to capture extended sequences with a smaller number of layers and minimal computational costs.

#### Temporal Gated Convolution (TGC)

Gating mechanisms for TCNs extract complex temporal dependencies, manage information flow [48], and increase the ability to manage long sequences, thereby improving model performance. Thus, the proposed architecture incorporates a gating mechanism into the TCN to form a gated TCN by constructing two TCN models, $TCN_{A}$ and $TCN_{B}$. $TCN_{B}$ is employed to produce gating signals, which are then dot-multiplied by $TCN_{A}$ for creating a TGC as shown in Figure 3b. The equation of TGC is as follows:

(7) $$X_{TGC}=TCN_{A}\left(X\right)⊙\sigma \left(TCN_{B}\left(X\right)\right)$$

where σ refers to the sigmoid function, which is used to emphasize strong relationships and filter out the weaker ones. The symbol ⊙ represents the element-wise product, $X_{TGC}\in R^{T\times N\times d}$ signifies the output produced by the TGC, and *d* denotes the embedding dimension.

TCM utilizes three TGCs: hourly TGC, daily TGC, and weekly TGC. These TGCs capture temporal dependencies across different scales by processing multi-scale temporal input segments $(X_{h}$, $X_{d}$, $X_{w}$).

The equation of hourly TGC is as follows:

(8) $$X_{HTGC}^{l}=\left(W_{th}⋆X_{h}^{l-1}+b_{t}\right)⊙\sigma \left(W_{th}⋆X_{h}^{l-1}+c_{t}\right)$$

The equation of daily TGC is as follows:

(9) $$X_{DTGC}^{l}=\left(W_{td}⋆X_{d}^{l-1}+b_{t}\right)⊙\sigma \left(W_{td}⋆X_{d}^{l-1}+c_{t}\right)$$

The equation of weekly TGC is as follows:

(10) $$X_{WTGC}^{l}=\left(W_{tw}⋆X_{w}^{l-1}+b_{t}\right)⊙\sigma \left(W_{tw}⋆X_{w}^{l-1}+c_{t}\right)$$

where $X_{h}^{l-1}$, $X_{d}^{l-1}$, and $X_{w}^{l-1}$ refer to the output generated by the (l−1)th block, ⋆ signifies the convolution operator, $W_{th},W_{td},W_{tw},b_{t}$, and $c_{t}$ are learnable parameters, σ indicates the sigmoid function, and ⊙ represents the element-wise product. TCM ultimately produces a final outcome by concatenating the three output temporal vectors $X_{HTGC}^{l}$, $X_{DTGC}^{l}$, and $X_{WTGC}^{l}$, and a linear transformation is then applied to align the output channels as demonstrated in Equation (11).

(11) $$\left(X_{temp}^{l}\right)=Concat\left(X_{HTGC}^{l},X_{DTGC}^{l},X_{WTGC}^{l}\right)⊙M$$

where $\left(X_{temp}^{l}\right)\in R^{T\times N\times d}$ represents the output generated by TCM. ⊙ is the element-wise product, and *M* denotes the masking matrix.

### 4.4. Multi-Graph Spatial Correlation Modeling (MGSCM)

**Figure 4:**
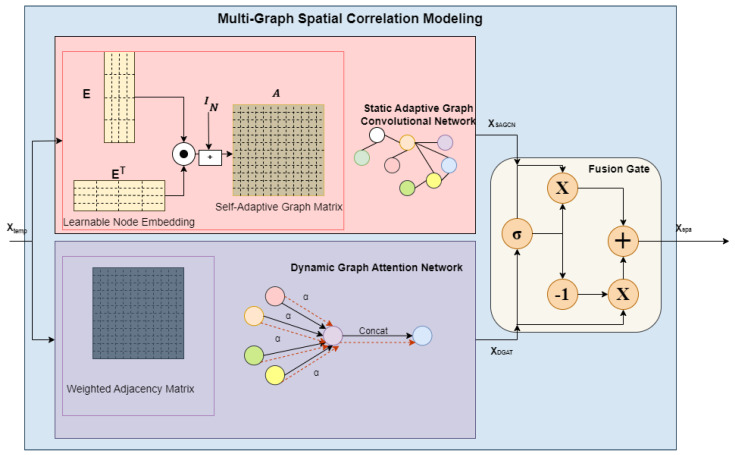
An architectural diagram of MGSCM.

MGSCM comprises two parallel graph networks: a Static Adaptive Graph Convolutional Network (SAGCN) and a Dynamic Graph Attention Network (DGAT), as shown in Figure 4. These networks are designed to adaptively and dynamically identify complex spatial relationships among sensor nodes representing different regions in real-world scenarios.

#### 4.4.1. Static Adaptive Graph Convolutional Network (SAGCN)

The proposed architecture leverages the SAGCN to represent stable spatial relationships among sensor nodes, independent of geographic distance. This is achieved through the utilization of the GCN. Traditional GCNs rely on a fixed, pre-defined graph structure. However, this static graph may not accurately represent spatial dependencies, as connections between nodes are solely based on proximity. This limitation can introduce substantial biases, hindering the effectiveness of the existing GCN in capturing the underlying spatial relationships [11,16].

To address the issue, a self-adaptive graph matrix *A* is constructed through continuous optimization during training via end-to-end modeling with stochastic gradient descent and learnable parameters, which allow the model to discover hidden spatial relationships independently. It is accomplished through the random initialization of a learnable node embedding dictionary $E\in R^{N\times d_{1}}$ for each node, where $d_{1}$ indicates the node embedding matrix’s dimensions. Through the node embedding multiplication, the spatial correlations among all node pairs can be assessed.

The process for constructing the self-adaptive graph matrix *A* is presented in the following equation:

(12) $$A=softmax(I_{N}+ReLU\left(tanh\left(EE^{T}\right)\right))$$

where $I_{N}$ represents the identity matrix. ReLU() and tanh() are non-linear activation functions. The softmax() is employed for graph matrix normalization.

Given the effectiveness of the GCN in extracting features from graph data, it was employed to extract the intricate spatial dependencies among nodes. The graph convolution operation in the SAGCN can be defined by combining the self-adaptive graph matrix *A* with the GCN, as expressed below:

(13) $$X_{SAGCN}^{l}=AX_{temp}^{l}\Theta$$

where $X_{SAGCN}^{l}\in R^{T\times N\times d}$ represents output generated by the SAGCN, and $\Theta \in R^{d\times d}$ refers to the convolutional kernel.

#### 4.4.2. Dynamic Graph Attention Network (DGAT)

In reality, spatial correlations among nodes evolve over time and are influenced by their neighboring nodes. For example, sudden accidents, construction, and road maintenance can lead to unexpected vehicle volume changes in adjacent areas. These dynamic factors can significantly alter the spatial relationships among road network nodes. GAT is employed to capture these dynamic spatial relationships. GAT leverages an attention mechanism to determine the hidden representation of each node in the graph, learns the relative weights of neighboring nodes, and aggregates their spatial features. The attention weights adjust according to data changes, introducing dynamic spatial correlations to the network.

**Figure 5:**
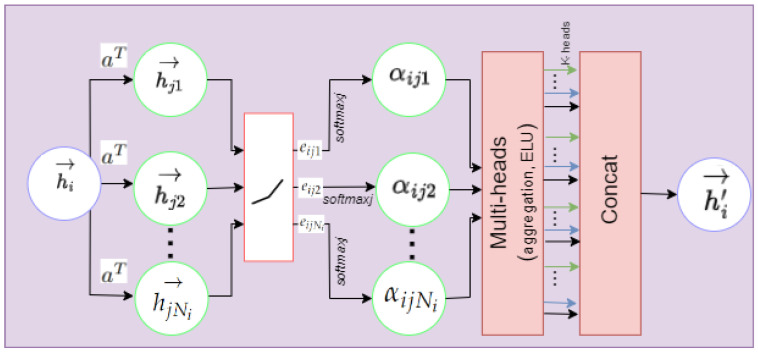
A structure diagram of GAT.

The inputs required for DGAT are the weighted adjacency matrix $A\in R^{N\times N}$ and sensor node feature set at time step *t* in the $X_{temp}^{l}$, denoted as ht={h1t→,h2t→………Nt→}, where $\vec{h^{t}}\in R^{d}$, and *N* refers to sensor node numbers. Suppose the sensor node features for node *i* and node *j* are $\vec{h_{i}^{t}}$, and $\vec{h_{j}^{t}}$, and $j\in N_{i}$, where $N_{i}$ indicates the set of neighboring nodes associated with node *i* as shown in Figure 5. The process for computing the attention score $e_{ij}^{t}$ between pairs of sensor nodes (*i* and *j*) at a time step *t* is demonstrated in Equation (14):

(14) $$e_{ij}^{t}=LeaklyReLU(a^{T}(W\vec{h_{i}^{t}}||W\vec{h_{j}^{t}}))$$

where *a* specifies the function used to compute the attention score,  $\cdot^{T}$ is the transposition, $W\in R^{d^{\prime }\times d}$ represents the weight matrix for all sensor nodes, || signifies the concatenation operation, and LeakyReLU denotes the non-linear activation function to filter out less significant relationships. The attention coefficient $\alpha_{ij}^{t}$ is obtained by normalizing the attention score $e_{ij}^{t}$ for all the neighboring sensor nodes *j* relative to sensor node *i* as follows:

(15) $$\alpha_{ij}^{t}=softmax\left(e_{ij}^{t}\right)=\frac{exp(e_{ij}^{t})}{\sum_{k\in N_{i}}exp(e_{ik}^{t})}$$

where $e_{ij}^{t}$ denotes the attention score between sensor nodes ji, and softmax() is a non-linear activation function.

Lastly, the output feature $\vec{h_{i}^{\prime t}}$ can be obtained by a multi-head graph attentional layer that aggregates features produced by multi-head attention through the summation after applying the non-activation ELU function to reduce negative values as follows:

(16) $$\vec{h_{i}^{\prime t}}=Concat(ELU(\sum_{j\in N_{i}}\alpha_{ij}^{k}W^{k}\vec{h_{j}^{t}}))$$

where *k* signifies head numbers. For head *k*, $W^{k}$ denotes the transformation parameter matrix $\in R^{d^{\prime }\times d}$. $\vec{h_{i}^{\prime t}}\in R^{d}$ represents the GAT output for sensor node *i* at time step *t* in $X_{temp}^{l}$. Similarly, each node undergoes the attention process to derive its output representation. Equation (17) uniformly demonstrates the steps using matrix calculations:

(17) $$X_{DGAT}^{l}=\left(\left(A⊙M\right)h_{t}^{\left(l\right)}W\right)$$

*A* is the weighted adjacency matrix created according to distance relationships among sensor nodes. $M\in R^{N\times N}$ is a matrix of dynamic attention factors. $h_{t}^{\left(l\right)}$ is the input of the $l^{th}$ block at time step *t*, comprising a collection of features from the sensor nodes. The final output vector for $l^{th}$ block of DGAT is $X_{DGAT}^{l}$$\in R^{T\times N\times d}$.

#### 4.4.3. Fusion Gate

For harnessing the strengths of both static and dynamic graph networks within (SAGCN) and (DGAT), a fusion gate was developed to effectively integrate the composite spatial dependencies. The operational details of the fusion gate mechanism are as follows:

(18) $$X_{spa}^{l}=gate⊙X_{SAGCN}^{l}+\left(1-gate\right)⊙X_{DGAT}^{l}$$

with

(19) $$gate=\sigma (X_{SAGCN}^{l}W_{g1}+X_{DGAT}^{l}W_{g2}+b_{g})$$

where gate is a weight vector that dynamically regulates the static and dynamic flow of spatial relationships in every sensor node. σ signifies a sigmoid activation function. $W_{g1},W_{g2}$, and $b_{g}$ denote parameters. The output yielded by MGSCM, denoted as $X_{spa}^{l}\in R^{T\times N\times d}$, represents the learned spatial features extracted from the input data.

### 4.5. Spatio-Temporal Self-Attention Modeling (STSAM)

**Figure 6:**
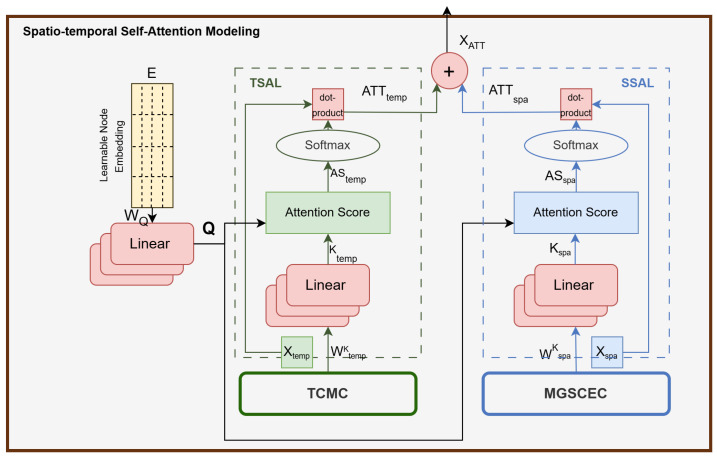
A diagram for STSAM.

Spatio-temporal data exhibit variability across both the spatial and temporal dimensions. To adaptively capture these variations, STSAM is introduced as illustrated in Figure 6. STSAM comprises two distinct layers: a Temporal Self-Attention Layer and a Spatial Self-Attention Layer. Initially, a learnable node embedding dictionary, $E\in R^{N\times d_{1}}$, is employed to demonstrate the characteristics of an arbitrary node *v* in a high-dimensional space. Notably, the same node embedding *E* is utilized in MGSCM to ensure a consistent high-dimensional representation of nodes throughout the model, thereby reducing the number of parameters. Subsequently, a node-level self-attention mechanism is applied.

#### 4.5.1. Temporal Self-Attention Layer (TSAL)

Traffic conditions at different time steps are influenced by each other, and these relationships can change dynamically. TSAL is utilized to adaptively capture long-term dynamic temporal relationships with a consideration of real-node characteristics. This layer takes $X_{temp}^{l}$, the output of TCM, as an input for the temporal dimension. Specifically, Query: Q, Key: K, and Value: V matrices for an arbitrary node v∈V are computed as follows:

(20) $$Q^{v}=E^{v}W_{Q},K_{temp}^{v}=X_{temp}^{l}W_{temp}^{K},V_{temp}^{v}=X_{temp}^{l}$$

where $W_{Q}$ and $W_{temp}^{K}\in R^{d\times d^{\prime \prime }}$ are learnable projection matrices, while $d^{\prime \prime }$ represents the dimensions of the matrices. In the next step, matrix products are computed and subsequently normalized to derive the attention scores for node *v* in each time step:

(21) $$AS_{temp}^{v}=\frac{Q^{v}{\left(K_{temp}^{v}\right)}^{T}}{\sqrt{d^{\prime \prime }}}$$

The output of the TSAL is computed as follows:

(22) $$ATT_{temp}\left(Q^{v},K_{temp}^{v},V_{temp}^{v}\right)=Softmax\left(AS_{temp}^{v}\right)V_{temp}^{v}$$

where softmax() is employed to normalize weight scores, ensuring they form a probability distribution.

#### 4.5.2. Spatial Self-Attention Layer (SSAL)

Within the spatial context, traffic situations at different areas influence one another, with these interactions changing dynamically. The SSAL is employed to effectively capture the evolving correlations between nodes with a consideration of real-node characteristics. This layer takes $X_{spa}^{l}$, the output of MGSCM, as an input for the spatial dimension. Specifically, Query: Q, Key: K, and Value: V matrices for an arbitrary node v∈V at time step *t* can be calculated as follows:

(23) $$Q^{v}=E^{v}W_{Q},K_{spa}^{v}=X_{spa}^{l}W_{spa}^{K},V_{spa}^{v}=X_{spa}^{l}$$

where $W_{Q}$ and $W_{spa}^{K}\in R^{d\times d^{\prime \prime }}$ are learnable projection matrices, while $d^{\prime \prime }$ represents the dimensions of the matrices. Subsequently, matrix products are computed and then normalized to derive the attention scores for node *v* at time step *t* using the following equation:

(24) $$AS_{spa}^{v}=\frac{Q^{v}{\left(K_{spa}^{v}\right)}^{T}}{\sqrt{d^{\prime \prime }}}$$

Finally, the output of the SSAL can be calculated as follows:

(25) $$ATT_{spa}\left(Q^{v},K_{spa}^{v},V_{spa}^{v}\right)=Softmax\left(AS_{spa}^{v}\right)V_{spa}^{v}$$

#### 4.5.3. Spatio-Temporal Self-Attention Concatenation

The outputs of Equations (22) and (25) are concatenated to obtain the final output of STSAM, denoted as $X_{ATT}^{l}$:

(26) $$X_{ATT}^{l}=Concat\left(ATT_{temp},ATT_{spa}\right)$$

By incorporating STSAM, the network focuses on the most significant information and effectively captures both dynamic and long-term spatio-temporal correlations, thereby enhancing traffic prediction accuracy.

### 4.6. Skipping Layer

Skip connections are employed by applying a standard convolution layer to each output from the AMST block and connecting them to the output layer. It transforms the output, $X_{ATT}$, into $X_{skip}\in R^{T\times N\times d_{skip}}$, where $d_{skip}$ is the dimension of skip. This transformation can be carried out as follows:

(27) $$X_{skip}^{l}=Conv\left(X_{ATT}^{l}\right)$$

These connections are crucial for capturing spatial dependencies across various temporal scales. By incorporating these skip connections, the model improves its ability to generate accurate traffic flow predictions by retaining critical information from previous layers, bypassing intermediate ones, and connecting to the final layers. The outputs from all skip connections are aggregated via summation, as described in Equation (28), yielding the final result denoted as $X_{final}\in R^{T\times N\times d_{skip}}$:

(28) $$X_{final}=\sum_{i=1}^{l}X_{skip}\left(i\right)$$

### 4.7. Output Layer

To predict the traffic flow for multi-steps, the output layer applies two standard 1 × 1 convolution layers to generate the final prediction as follows:

(29) $$\hat{X}=Conv^{2}\left(ReLU\left(Conv^{1}\left(X_{final}\right)\right)\right)$$

where $Conv^{2}$ and $Conv^{1}$ are two standard convolutional layers that transform the skip dimensions of $X_{final}$ and time steps, respectively. ReLU() is the non-linear activation function, and $\hat{X}\in R^{T_{p}\times N\times F}$ denotes the prediction result for time steps $T_{p}$.

## 5. Experiments and Discussion

This section presents the experimental results of the ASTAM, comparing its performance to baseline models across four real-world datasets using various evaluation metrics for a comprehensive assessment. Ablation studies were conducted to evaluate the contribution of each component, along with additional tests on parameter sensitivity and the effectiveness of the self-adaptive matrix.

### 5.1. Datasets

To assess the predictive performance of the ASTAM, extensive tests were conducted using four publicly available real-world traffic datasets that were collected by the Caltrans Performance Measurement System, specifically PeMS03, PeMS04, PeMS07, and PeMS08 [49]. These datasets were obtained by utilizing sensors located in California in the United States of America. Every 30 s, the sensors collected data, which were then aggregated every five minutes to create a data point. Table 1 shows the details of these datasets.

### 5.2. Settings

The experiments were conducted on a server configured with one NVIDIA Tesla A100 GPU with 40 GB of memory. The ASTAM was developed with Python 3.9.7 and PyTorch. All the datasets were divided into sets for training, validation, and testing with ratios 6:2:2. Table 2 shows the total number of samples in addition to the training and test samples. In the prediction process, the previous time steps were set to twelve for every segment, forming a time series used to predict the subsequent twelve time steps. This configuration aims to forecast traffic flow over a one-hour period into the future, denoted by the symbol $T_{p}=12$.

The ASTAM was trained on each dataset five times, and the average results were used. Throughout the training phase, batch size was assigned a value of 32, with a learning rate of 0.0001. The embedding dimension *d* was configured as 64, the kernel size and layer numbers of the TCN were set to 3 and 4, respectively.

### 5.3. Evaluation Metrics

Throughout the studies, the prediction accuracy of the ASTAM was assessed with three metrics: Mean Absolute Error (MAE), Root Mean Squared Error (RMSE), and Mean Absolute Percentage Error (MAPE).

MAE calculates the absolute difference between the actual and predicted values to determine the prediction accuracy. It is calculated as follows:

(30) $$MAE\left(x,\hat{x}\right)=\frac{1}{N}\sum_{i=1}^{N}\left|x_{i}-{\hat{x}}_{i}\right|$$

RMSE calculates the variation between the actual and predicted values, indicating the extent to which the model fits the data. RMSE is particularly sensitive to outliers. It is calculated as follows:

(31) $$RMSE\left(x,\hat{x}\right)=\sqrt{\frac{1}{N}\sum_{i=1}^{N}{\left(x_{i}-{\hat{x}}_{i}\right)}^{2}}$$

MAPE offers insight into the proportional error comparing the actual and predicted results by expressing the percentage variation between the actual and predicted values. It is calculated as follows:

(32) $$MAPE\left(x,\hat{x}\right)=\frac{1}{N}\sum_{i=1}^{N}\left|\frac{x_{i}-{\hat{x}}_{i}}{x_{i}}\right|\times 100\%$$

where $x_{i}$ represents the true value. ${\hat{x}}_{i}$ represents the predicted result for the $i^{th}$ time sample, with *N* representing the number of samples.

### 5.4. Baselines

To validate the ASTAM, comparisons with representative baseline approaches were performed. The selected baselines include traditional prediction techniques, neural network models, and spatio-temporal deep learning techniques, including GNNs and attention-based approaches. Table 3 summarizes the baseline approaches.

### 5.5. Experimental Results and Analysis

This study aims to evaluate the extent to which the proposed ASTAM architecture improves the accuracy of traffic flow prediction. The results obtained from experiments are discussed in this section to address this question. Table 4 showcases the results of the proposed ASTAM architecture comparing its performance against the baseline approaches for next-hour predictions across four datasets.

The experimental results reveal that the proposed ASTAM architecture outperformed all baseline approaches across all metrics on the four datasets, demonstrating its superior predictive performance. Specifically, when compared to the best baseline approach, DSTAGNN, the proposed ASTAM architecture demonstrated reductions on the PeMS03 dataset of 16.57% in RMSE, 4.94% in MAE, and 6.60% in MAPE. Similarly, on the PeMS04 dataset, the ASTAM achieved reductions of 11.18% in RMSE, 1.23% in MAE, and 5.46% in MAPE. On the PeMS07 dataset, the ASTAM achieved reductions of 12.69% in RMSE, 5.87% in MAE, and 9.97% in MAPE. On the PeMS08 dataset, the ASTAM demonstrated reductions of 12.67% in RMSE, 8.28% in MAE, and 3.8% in MAPE.

Overall, spatio-temporal deep learning techniques, including GNNs and attention-based approaches (including DCRNN, STGCN, GWN, ASTGCN, STSGCN, AGCRN, Z-GCNET, DSTAGNN, and ASTAM) outperformed traditional techniques, like VAR, ARIMA, SVR, and neural network models such as FC-LSTM and TCN. This is likely due to the fact that traditional techniques and neural network models primarily focus on temporal dependencies, neglecting the spatial correlations within traffic data. Accurate traffic flow modeling requires the consideration of both temporal and spatial relationships.

Spatio-temporal deep learning techniques such as DCRNN, STGCN, and GWN typically outperform neural network models like FC-LSTM and TCN. This advantage stems primarily from the ability of GNN-based models to effectively extract spatial relationships from traffic data. However, DCRNN, STGCN, and GWN rely on pre-defined distance-based graphs. In contrast, the ASTAM surpasses these models by utilizing a self-adaptive graph matrix, which eliminates the need for pre-defined graphs and accurately captures the real spatial dependencies within the data. The ASTAM reduced the average MAE by 19.9%, 17.8%, and 24.7%; the average RMSE by 23.3%, 22.4%, and 29.5%; and the average MAPE by 25.6%, 20%, and 28.8%, compared with DCRNN, STGCN, and GWN, respectively, across the four datasets.

Additionally, the ASTGCN employs a single convolution layer, which restricts its ability to capture temporal dependencies as it relies solely on information from the neighboring time segment, disregarding variations across different time segments. The STSGCN, on the other hand, employs learnable temporal and spatial embedding matrices that effectively capture local spatio-temporal variations. However, the STSGCN does not address the dynamic spatio-temporal relationships between nodes. The proposed model, ASTAM, incorporates multi-gated modules that can capture long-term dynamic temporal correlations, leading to improved prediction results. The ASTAM on the four datasets achieved 16.8% and 14.2% improvements in the average MAE, 21.2% and 19.6% improvements in the average RMSE, and 22.4% and 15.7% improvements in the average MAPE, compared to the ASTGCN and STSGCN.

AGCRN and Z-GCNET employ GRU for temporal learning; however, these models may still be limited in extracting complex, dynamic, and long-term temporal relationships. For spatial dependency learning, AGCRN and Z-GCNET adopt distinct approaches: AGCRN uses learnable node parameters to adaptively model spatial dependencies, whereas Z-GCNET employs time-aware convolutions for capturing only the topological features of the data. In contrast, the proposed model, ASTAM, excels in the spatial dimension by not only considering adaptive static node relationships but also learning dynamic relationship changes, resulting in improved predictive performance. Compared to AGCRN and Z-GCNET across the four datasets, ASTAM reduced the average MAE by 7.3% and 7.5%, the average RMSE by 15.7% and 14.3%, and the average MAPE by 8.14% and 10.37%, respectively.

The DSTAGNN employs an attention mechanism to enhance its ability to capture dynamic spatial correlations and utilizes multi-gated modules to extract temporal relationships. However, the multi-gated modules struggled to effectively capture dynamic temporal relationships. In contrast, the ASTAM employs self-attention mechanisms for both spatial and temporal relationships, leading to improved performance.

To elaborate further, most of the studies mentioned employ separate modules to sequentially capture spatio-temporal correlations. In contrast, the ASTAM leverages a unified approach by incorporating a self-attention mechanism to simultaneously capture both spatial and temporal dependencies. This innovative approach allows the ASTAM to dynamically calculate the correlation strengths between nodes and time steps. By emphasizing the most influential nodes and time steps on the current traffic state, the architecture adaptively captures dynamic and long-term spatio-temporal information.

**Figure 7:**
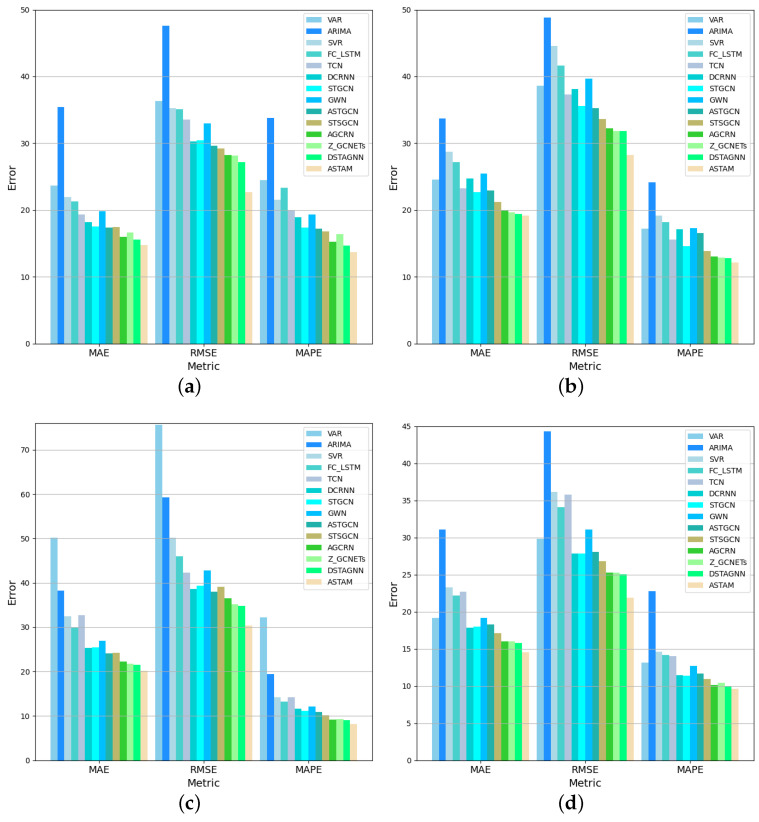
ASTAM versus different baselines for (**a**) PeMS03, (**b**) PeMS04, (**c**) PeMS07, and (**d**) PeMS08.

In summary, most of the previous models have employed a variety of graph structures to learn spatio-temporal traffic dependencies, often integrating them with TCN- or RNN-based techniques. While these approaches have shown promise, the ASTAM stands out by operating effectively across diverse traffic scenarios without needing pre-defined graphs. The ASTAM’s innovative approach involves extracting intricate spatial dependencies within traffic flow through a combination of static and dynamic graph modeling. Additionally, the model utilizes multi-temporal gated convolution to efficiently capture temporal dependencies across different periods. Finally, self-attention plays an essential role in capturing long-term dynamic spatio-temporal variations, contributing significantly to the model’s overall performance. As demonstrated in Figure 7, the ASTAM consistently achieves the highest performance across all metrics for the four datasets PeMS03, PeMS04, PeMS07, and PeMS08. These results support the hypothesis that the ASTAM improves traffic flow prediction accuracy by effectively capturing complex spatio-temporal dependencies.

### 5.6. Ablation Study

The experiments conducted on four datasets aimed to reinforce the validation of each component’s importance in the ASTAM. Specifically, the removal of any individual component resulted in a reduction in prediction accuracy that indicates the crucial role of each component in capturing essential information and ultimately enhancing overall performance. In ablation study experiments, the prediction time step was fixed at twelve, while all other parameters remained consistent with the main results. The experiments involved five different variants as follows:

- w/o TCM: TCM is removed from the ASTAM.
- w/o SAGCN: SAGCN is excluded from ASTAM.
- w/o DGAT: DGAT is eliminated from ASTAM.
- w/o Fusion: Fusion gate is omitted from ASTAM.
- w/o STSAM: STSAM is canceled from ASTAM.

**Figure 8:**
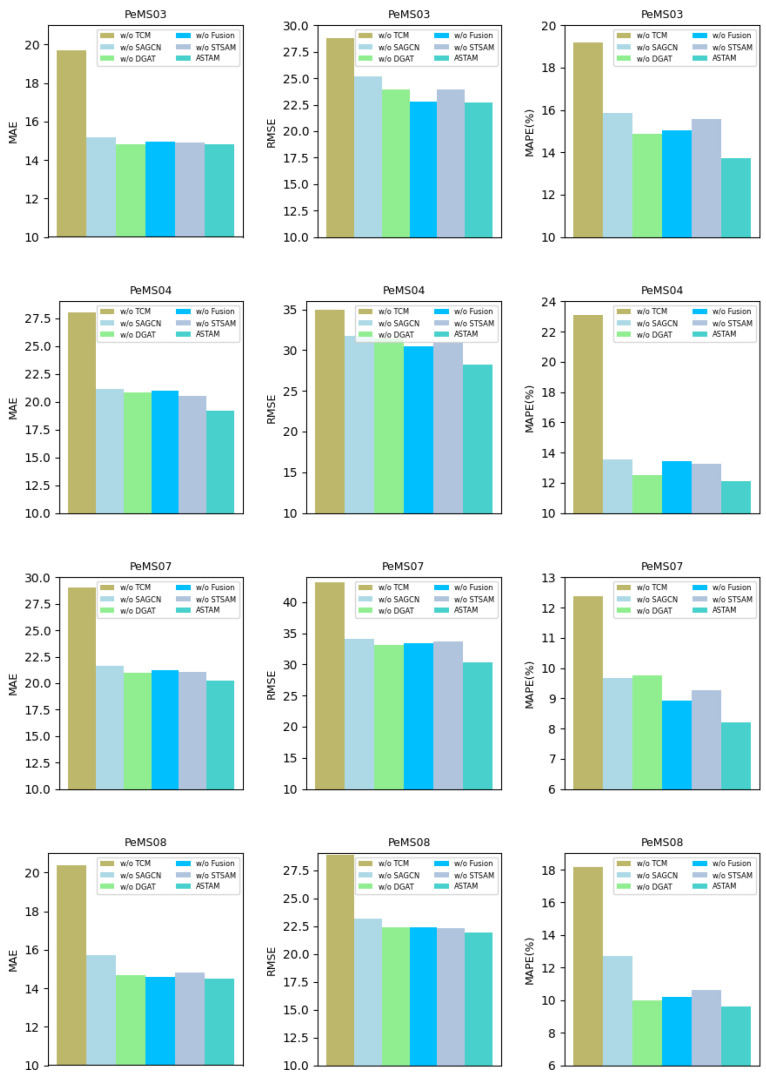
The ablation experiments conducted on all datasets.

The findings from the ablation studies, detailed in Table 5 and visualized in Figure 8, offer insights into the performance of the model when various components are removed. The studies demonstrate that the ASTAM achieved the best results, indicating that each individual component had a significant impact on overall performance. In addition, the variant (w/o TCM) performed the worst across all datasets, demonstrating the significant effect of the TCM on enhancing prediction accuracy. The variant (w/o SAGCN) ranked the second-lowest, which indicates the model heavily depends on capturing static hidden spatial dependencies. The impact of the remaining components varied across datasets as a result of the differing intrinsic patterns existing within these datasets. Overall, each component within the ASTAM significantly contributed to enhancing the prediction.

According to the ablation studies, the variant (w/o TCM) evidently obtained the lowest prediction performance among all variants on all datasets compared to the ASTAM. For instance, in terms of MAE, its performance decreased by 24.8% and 31.4% for PeMS03 and PeMS04, respectively, whereas it dropped by 30.3% and 28.9% for PeMS07 and PeMS08, respectively. These reductions demonstrate the significance of TCM in enhancing the performance of the ASTAM by effectively capturing temporal correlations within traffic data.

For the variant (w/o SAGCN), the prediction performance was notably worse. In terms of MAE, its performance decreased by 2.63%, 9%, 6.4%, and 7.6% for PeMS03, PeMS04, PeMS07, and PeMS08, respectively, compared to the ASTAM. These results demonstrate the crucial role of static hidden spatial dependencies in enhancing prediction, as the performance of the ASTAM declined when relying solely on dynamic influences. This underscores the importance of incorporating a self-adaptive matrix within the SAGCN.

Moreover, the variant (w/o DGAT) showed lower prediction accuracy across various datasets compared with the ASTAM. For instance, in terms of MAE, the performance declined relative to the ASTAM by 0.13%, 7.69%, 3.9%, and 1.36% for PeMS03, PeMS04, PeMS07, and PeMS08, respectively. This indicates that DGAT can effectively improve prediction results by considering the influence of neighboring node relationships and dynamically adjusting spatial correlations over time.

Furthermore, the SAGCN and DGAT within the MGSCM component each offer distinct advantages that can contribute to more accurate predictions. Notably, the SAGCN appears to have a greater impact than the DGAT. The fusion of these components positively influences the predictive performance of the ASTAM. The ASTAM achieved improvement in MAE when compared with (w/o Fusion) by 1.14%, 9.3%, 4.9%, and 0.68% for PeMS03, PeMS04, PeMS07, and PeMS08, respectively.

Additionally, the predictive performance for the variant (w/o STSAM) was worse than the ASTAM. The results demonstrate the significance of considering node characteristics and the self-attention role in extracting the long-term spatio-temporal correlation, which ultimately contributes to improved performance in the ASTAM. For example, the ASTAM achieved a reduction in RMSE compared to the variant (w/o STSAM) by 0.67%, 6.3%, 4.26%, and 2.02% for PeMS03, PeMS04, PeMS07, and PeMS08, respectively.

### 5.7. Parameter Sensitivity Study

For evaluating the impact of main hyper-parameters on the ASTAM’s predictive performance, sensitivity experiments were conducted on the number of layers in the TCN and the data embedding dimension *d* using the PeMS04 dataset.

**Figure 9:**
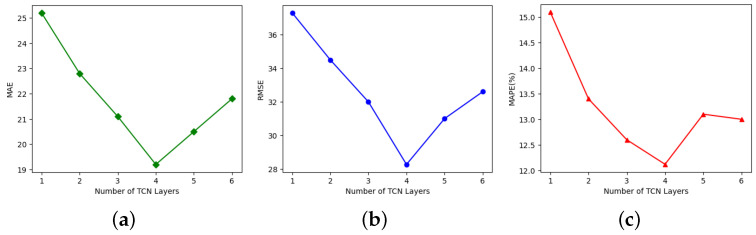
Influence of varying the number of layers in TCN on (**a**) MAE, (**b**) RMSE, and (**c**) MAPE.

This study evaluated the influence of varying the number of layers in the TCN by testing values of {1, 2, 3, 4, 5, 6}. The results, based on MAE, RMSE, and MAPE metrics, are shown in Figure 9. Initially, the prediction performance improved significantly with an increase in the number of layers. The best achievable performance was observed when the TCN layer was determined to be 4. However, further increasing the number of layers resulted in a decline in overall prediction accuracy.

**Figure 10:**
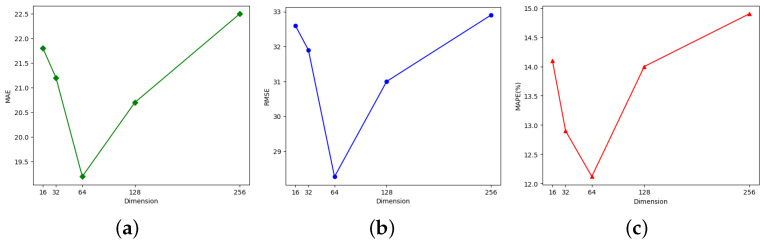
Effect of the various embedding dimensions on (**a**) MAE, (**b**) RMSE, and (**c**) MAPE.

For the data embedding dimension *d*, as illustrated in Figure 10, the optimal prediction results for the data embedding dimension *d* were obtained when it was set to 64. Overall, the pattern indicates that prediction performance improved by increasing the embedding dimension, peaking at 64, and then declined with additional increases. This occurred because a larger embedding dimension initially enhanced the model’s capacity to capture critical information. Once the optimal point was surpassed, increasing the embedding dimension further resulted in extended training time and reduced prediction accuracy due to over-fitting.

### 5.8. Effectiveness of Self-Adaptive Matrix

**Figure 11:**
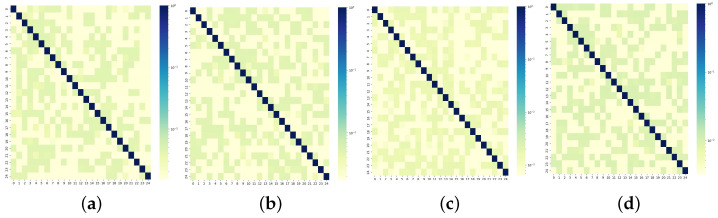
Heatmap representation of the self-adaptive matrix for (**a**) PeMS03, (**b**) PeMS04, (**c**) PeMS07, and (**d**) PeMS08.

**Figure 12:**
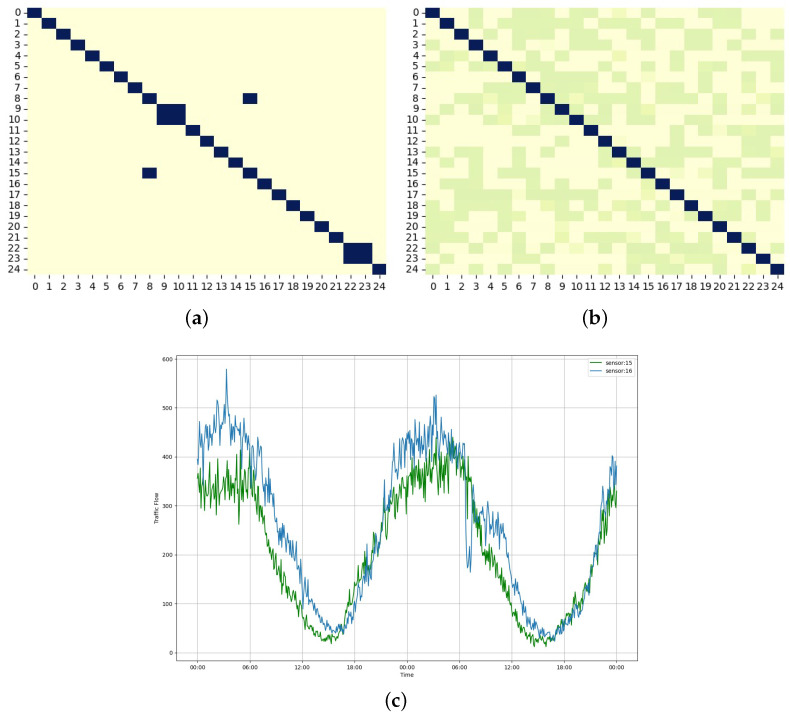
Effectiveness of the self-adaptive matrix. (**a**) Pre-defined matrix; (**b**) self-adaptive matrix; (**c**) 48 h traffic flow on sensor pairs 15–16.

To illustrate the effectiveness of the self-adaptive matrices within the SAGCN generated through iterative training, the top 25 sensors in each of the four datasets are visualized as shown in Figure 11. The correlations between selected sensor pairs are presented for both pre-defined and self-adaptive matrices of the PeMS04 dataset to further illustrate the advantages of employing an SAGCN as shown in Figure 12.

A self-adaptive matrix can learn hidden correlations between sensor pairs regardless of their physical distance. For example, sensors 15 and 16 appear unrelated in the pre-defined matrix, which relies solely on geographic distance, as shown in Figure 12a. However, they, in fact, exhibit similar traffic patterns over time, as demonstrated by the visualized traffic flow curves in Figure 12c. The self-adaptive matrix can effectively capture these hidden spatial correlations between these sensors, as shown in Figure 12b. This demonstrates the limitations of the pre-defined matrix in reflecting actual spatial dependencies between sensors, as distance alone may not guarantee strong correlations. These visualizations emphasize the effectiveness of the ASTAM in capturing hidden spatial dependencies between sensors using a self-adaptive matrix.

### 5.9. Visualization Analysis

**Figure 13:**
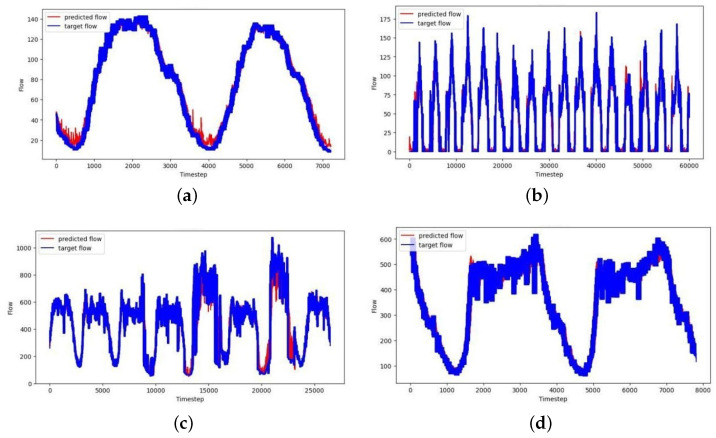
Traffic flow prediction result visualization of (**a**) PeMS03, (**b**) PeMS04, (**c**) PeMS07, and (**d**) PeMS08.

To comprehensively evaluate the predictive capabilities of the ASTAM, its predictions on the test set were compared with the actual traffic values through visualization. The visualization reveals that the prediction results show similar flow and trends compared to the actual traffic volumes during the predicted times, as shown in Figure 13. Even under challenging traffic conditions, the ASTAM delivered accurate predictions that corresponded closely to actual traffic volumes and effectively captured changes during significant traffic fluctuations, as demonstrated in Figure 13b. This confirms the ASTAM’s ability to effectively represent global traffic flow variations and capture diverse dependency features. Overall, the consistently strong performance of the ASTAM across various datasets underscores its reliability and robustness.

## 6. ASTAM’s Potential Real-World Applications

The proposed ASTAM architecture not only contributes to the advancement of traffic flow prediction but can also be adaptable to a variety of real-world applications such as traffic congestion, estimating travel time, and urban infrastructure construction.

To elaborate more, the ASTAM can help travelers to plan their journeys more efficiently while supporting traffic authorities in optimizing resource allocation, enhancing signal coordination, and implementing effective congestion mitigation strategies.

Moreover, the ASTAM can be integrated with urban ITSs to strengthen real-time traffic monitoring, improve traffic flow management, and support optimized scheduling. These capabilities provide policymakers with valuable insights for future transportation infrastructure development.

To sum up, the ASTAM’s potential applications across various tasks can contribute to minimizing the environmental impact of transportation and fostering urban sustainability through advanced technological solutions.

## 7. Conclusions

This research introduces a new traffic flow prediction architecture known as the ASTAM. The ASTAM employs multi-temporal gated convolution in a TCM to effectively capture the non-linear temporal correlations across different periods inherent within traffic data. Additionally, an MGSCM is introduced that combines static and dynamic multi-graph networks to model complex spatial correlations. Finally, an STSAM is incorporated into the architecture to adaptively capture long-term spatio-temporal variations. Empirical evaluations were carried out on four real-world datasets to assess the superiority and effectiveness of the proposed architecture in addressing traffic flow prediction challenges. According to the experimental results, the proposed architecture demonstrated a substantial improvement in traffic flow prediction performance across all four public datasets and evaluation metrics.

The proposed architecture has certain limitations that require further investigation. Firstly, the computational complexity of the architecture is relatively high, primarily due to the dot-product operations inherent in the spatio-temporal self-attention mechanism. Secondly, external factors such as weather, which can significantly influence traffic flow, are not modeled in the current architecture. Future research will focus on addressing these limitations by enhancing the model’s performance and optimizing its computational efficiency. The potential to extend the proposed architecture to real-world applications will be explored, alongside the integration of external conditions like weather data.

## Figures and Tables

**Table 1:** Overview of datasets.

Dataset	Sensors	Edges	Time Interval	Time Steps	Time Span
PeMS03	358	547	26,208	5 min	3 months
PeMS04	307	340	16,992	5 min	2 months
PeMS07	883	866	28,224	5 min	3 months
PeMS08	170	295	17,856	5 min	2 months

**Table 2:** Information on total, training, and test samples.

Dataset	Total Samples	Training Samples	Test Samples
PeMS03	26,208	15,726	5241
PeMS04	16,992	10,196	3398
PeMS07	28,224	16,936	5644
PeMS08	17,856	10,714	3571

**Table 3:** Baseline approaches.

Approach	Key Characteristics
**Vector Auto-Regression (VAR)** [50]	A statistical technique that models variables using previous values and relationships linearly.
**Autoregressive Integrated Moving Average Model (ARIMA)** [51]	A statistical technique that analyzes autocorrelation and uses differencing to predict time series patterns.
**Support Vector Regression (SVR)** [26]	An SVM-based approach that fits a hyperplane with a maximum margin and uses kernels for non-linear relationships.
**Fully Connected Long Short-Term Memory Model (FC-LSTM)** [52]	A neural network architecture that integrates dense layers and LSTM to capture temporal correlations.
**Temporal Convolutional Network (TCN)** [53]	A neural network architecture that uses causal convolutions with dilation for long-term temporal modeling.
**Diffusion Convolutional Recurrent Neural Network (DCRNN)** [9]	A spatio-temporal model that combines random walks for spatial graphs with GRUs for temporal extraction.
**Spatial–Temporal Graph Convolutional Network (STGCN)** [10]	A spatio-temporal model that incorporates the GCN for spatial and 1D convolution for temporal correlations.
**Graph WaveNet (GWN)** [16]	A spatio-temporal model that integrates diffusion convolutions and the GCN hierarchically for spatio-temporal data.
**Attention-Based Spatial–Temporal Graph Convolutional Network (ASTGCN)** [39]	A spatio-temporal model that integrates GCN, CNN, and attention mechanisms for dynamic spatio-temporal data.
**Spatio-temporal Synchronous Graph Convolutional Network (STSGCN)** [49]	A spatio-temporal model that combines GCN and synchronization mechanisms for local spatio-temporal patterns.
**Adaptive Graph Convolutional Recurrent Networks (AGCRNs)** [11]	A spatio-temporal model enhances the GCN by incorporating node-adaptive learning and data-adaptive graph construction, combined with GRUs for dependencies.
**Time-Aware Zigzags at Graph Convolutional Network (Z-GCNET)** [12]	A spatio-temporal model that uses a zigzag layer for time-aware GCNs and GRUs for temporal modeling.
**Dynamic Spatial–Temporal-Aware Graph Neural Networks (DSTAGNNs)** [40]	A spatio-temporal model that designs spatio-temporal attention and gated convolutions for dynamic extraction.

**Table 4:** Comparison of next-hour prediction performance across various models using PeMS datasets from real-world scenarios.

Model	PeMS03				PeMS04				PeMS07				PeMS08			
	MAE	RMSE	MAPE%		MAE	RMSE	MAPE%		MAE	RMSE	MAPE%		MAE	RMSE	MAPE%	
VAR	23.65	38.26	24.51		24.54	38.61	17.24		50.22	75.63	32.22		19.19	29.81	13.1	
ARIMA	35.41	47.59	33.78		33.73	48.8	24.18		38.17	59.27	19.46		31.09	44.32	22.73	
SVR	21.97	35.29	21.51		28.7	44.56	19.2		32.49	50.22	14.26		23.25	36.16	14.64	
FC-LSTM	21.33	35.11	23.33		27.14	41.59	18.20		29.98	45.94	13.2		22.2	34.06	14.2	
TCN	19.32	33.55	19.93		23.22	37.26	15.59		32.72	42.23	14.26		22.72	35.79	14.03	
DCRNN	18.18	30.31	18.91		24.7	38.12	17.12		25.3	38.58	11.66		17.86	27.83	11.45	
STGCN	17.55	30.42	17.34		22.7	35.55	14.56		25.38	39.34	11.21		18.02	27.83	11.4	
GWN	19.85	32.94	19.31		25.45	39.7	17.29		26.85	42.78	12.12		19.13	31.05	12.68	
ASTGCN	17.34	29.66	17.24		22.93	35.22	16.56		24.05	37.97	10.92		18.25	28.06	11.64	
STSGCN	17.48	29.21	16.78		21.19	33.65	13.9		24.26	39.03	10.2		17.13	26.80	10.96	
AGCRN	15.98	28.25	15.23		19.88	32.27	13.03		22.26	36.47	9.16		15.97	25.25	10.13	
Z-GCNET	16.64	28.15	16.39		19.67	31.86	12.9		21.79	35.15	9.27		16.03	25.28	10.39	
DSTAGNN	15.57	27.21	14.68		19.44	31.83	12.82		21.46	34.82	9.12		15.81	25.08	9.98	
**ASTAM**	**14.8**	**22.7**	**13.71**		**19.2**	**28.27**	**12.12**		**20.2**	**30.4**	**8.21**		**14.5**	**21.9**	**9.6**	

**Table 5:** Ablation study results.

Model	PeMS03				PeMS04				PeMS07				PeMS08			
	MAE	RMSE	MAPE%		MAE	RMSE	MAPE%		MAE	RMSE	MAPE%		MAE	RMSE	MAPE%	
w/o TCM	19.7	28.8	19.21		28	35	23.1		29	43.2	12.37		20.4	29.9	18.17	
w/o SAGCN	15.2	25.2	15.84		21.1	31.8	13.56		21.6	34.1	9.68		15.7	23.2	12.73	
w/o DGAT	14.82	23.9	14.87		20.8	31.4	12.5		21.02	33.1	9.76		14.7	22.4	9.96	
w/o Fusion	14.97	22.8	15.03		21	30.5	13.43		21.2	33.4	8.93		14.6	22.4	10.2	
w/o STSAM	14.9	23.8	15.56		20.5	30.9	13.26		21.1	33.7	9.27		14.8	22.3	10.65	
ASTAM	14.8	22.7	13.71		19.2	28.27	12.12		20.2	30.4	8.21		14.5	21.9	9.6	

## Data Availability

For this research, the datasets employed are publicly accessible real-world datasets.
